# Updates on Breast Cancer in Oman: Challenges and Promising Frontiers

**DOI:** 10.7759/cureus.85971

**Published:** 2025-06-13

**Authors:** Awf Al Khan, Noora Al Balushi

**Affiliations:** 1 Department of Pathology and Blood Bank, Sohar Hospital, Sohar, OMN

**Keywords:** breast cancer research, early detection of breast cancer, epidemiology, oman, risk factor, updates on breast cancer

## Abstract

Breast cancer is a significant public health concern in Oman, with increasing incidence rates over the past few decades. This literature review explores the epidemiology, risk factors, cultural influences, screening practices, treatment outcomes, research, and role of non-profit organizations in breast cancer awareness in Oman. The review highlights the unique challenges faced by Omani women, including late-stage diagnosis, cultural stigma, and limited awareness of screening programs. While efforts have been made to improve early detection and treatment, there is a need for more comprehensive public health strategies to address the growing burden of breast cancer in Oman. This review synthesizes recent studies to provide a comprehensive overview of the current state of breast cancer in Oman and suggests directions for future research and policy interventions.

## Introduction and background

Breast cancer is the most commonly diagnosed cancer in women worldwide, and Oman reflects this global trend, as shown in Table 1 [1]. In Oman, breast cancer accounts for a significant proportion of cancer-related morbidity and mortality [2].

**Table 1:** The most common cancer types in both genders according to the registered new cases in Oman in 2022

Rank	Cancer type	Number of new cases	Number of deaths
1	Breast	549	199
2	Colorectum	431	249
3	Non-Hodgkin lymphoma	265	136
4	Stomach	245	210
5	Leukemia	237	176
6	Prostate	217	86
7	Thyroid	212	26
8	Bladder	163	70
9	Lung	157	146
10	Liver	148	145

The incidence of breast cancer in Oman has been rising, with late-stage diagnosis being a major concern. According to the World Health Organization (WHO), the incidence rate of breast cancer was 34% while the mortality rate was 13.1% in Oman in 2022 [1].

In addition, Al-Sayegh et al. [3] studied the cancer incidence trends in Oman from 1996 to 2019. They found that breast cancer had an increasing trend, with an annual percent change (APC) of 4.19% [3]. Breast cancer age-standardized rate (ASR) increased from 13.6 to 35.4 [3].

This present review highlights breast cancer epidemiology, risk factors, cultural barriers, and healthcare interventions in Oman.

## Review

Epidemiology of breast cancer in Oman

Breast cancer is the leading cause of cancer-related deaths among Omani women. Data analyzed from 1996 to 2015 show that breast cancer accounts for approximately 21.2% of all cancer cases in Omani women [2]. By 2022, this percentage had increased to 34.9% [1]. The total number of new cases of female breast cancer has dramatically increased from 139 in 2010 (Table 2) [4] to 549 in 2022 (Table 1) [1]. The ASR of breast cancer in Oman and other Gulf Cooperation Council (GCC) countries is lower than in Western countries but is increasing rapidly due to lifestyle changes and improved cancer registration [5]. The average age at diagnosis in Oman is younger than in Western countries, with many cases occurring in women under 50 years of age [6,7].

**Table 2:** The number of new cases of female breast cancer in Oman from 2010 to 2020

Year	Number of new cases of female breast cancer
2010	139
2011	162
2012	158
2013	184
2014	177
2015	223
2016	279
2017	278
2018	299
2019	347
2020	266

Risk factors

The risk factors for breast cancer in Oman are similar to those globally but are influenced by regional and cultural contexts. The risk increases with age, and women are at higher risk than men [3]. In addition, a family history of breast cancer and genetic mutations such as BRCA1 and BRCA2 could be risk factors [8]. Other risk factors are early menarche, late menopause, and delayed childbirth [9]. Furthermore, low awareness of breast cancer symptoms and screening, coupled with cultural stigma, often leads to delayed diagnosis [10].

Cultural barriers to early detection

Cultural factors play a significant role in the late-stage diagnosis of breast cancer in Oman. Late-stage diagnosis is defined as the detection of breast cancer after the cancer cells have spread to other parts of the body. Many Omani women are reluctant to seek medical help due to modesty, fear of stigma, and lack of awareness [10]. Additionally, there is a misconception that breast cancer is incurable, which discourages women from participating in screening programs. Traditional beliefs and reliance on alternative medicine further delay diagnosis and treatment [10].

Screening practices

Breast cancer screening in Oman is primarily opportunistic rather than population-based. The Ministry of Health (MOH) has implemented a mammography screening program, but participation rates remain low due to a lack of awareness and cultural barriers [11]. Efforts to promote breast self-examination (BSE) and clinical breast examination (CBE) have been initiated, but their impact is limited by insufficient public education and outreach [11]. A recent study found that barriers to BSE are fear of a potential breast cancer diagnosis, the practical aspects of BSE, and embarrassment related to BSE [12]. Another study concluded that urgent action is needed to improve awareness of breast cancer and BSE among Omani women [9]. Healthcare professionals should persist in educating the public about breast cancer, while media outlets like television and newspapers must also play a role in spreading awareness. Furthermore, breast cancer education should be integrated into teacher training programs at schools and colleges to ensure broader outreach [9].

In 2025, the Department of Women and Child Health at the MOH launched the second edition of the National Early Detection and Screening for Breast Cancer Guideline. The first edition was launched in 2010. This recent guideline plays a critical role in organizing the screening services for breast cancer in Oman [13]. It highlights the aims and priorities of early detection, screening, and treatment of breast cancer, along with the current status of breast cancer in Oman. It explains the different types, pathology, and progression stages of the disease. Additionally, it details screening methods like clinical breast exams, self-examinations, and mammograms, targeting women aged 35-69 as well as those under 35, including pregnant and breastfeeding women [13]. The guideline also outlines the roles of the healthcare team responsible for screening, which consists of trained doctors and nurses, surgeons, radiologists, radiographers, heads of women and child health sections at the governorate level, the head of the women’s health section, and the directors of the women and child health department and radiology department [13].

New and emerging treatments

The treatment of breast cancer in Oman follows international guidelines, including surgery, chemotherapy, radiation therapy, and hormonal therapy. However, late-stage diagnosis often results in poorer outcomes and higher mortality rates [14]. Access to advanced treatments, such as targeted therapies, is limited by resource constraints and the high cost of care [15]. Despite these challenges, survival rates have improved in recent years due to better diagnostic and treatment facilities [16].

The Omani government and private sector are investing in cancer research centers and partnerships with global institutions to improve accessibility to cutting-edge treatments. Oman is making significant strides in breast cancer treatment through targeted therapies, immunotherapy, and personalized medicine. Continued investment in research, early detection, and healthcare infrastructure will further enhance patient outcomes [17].

Public health interventions and projects

To address the growing burden of breast cancer, Oman has implemented several public health initiatives. These include awareness campaigns, training programs for healthcare providers, and the establishment of cancer care centers [18]. Oman allocates approximately 2.6% of its gross domestic product (GDP) to healthcare, with expenditures increasing by 12.9% per year. However, exact spending on oncology services is difficult to determine due to several factors. First, cancer care involves multiple entities, including the MOH and the Ministry of Education. Second, diagnostic, screening, and treatment services are managed by different divisions within the MOH and by other health care organizations that partner with MOH in cancer care, such as the Sultan Qaboos Comprehensive Cancer Care and Research Center (SQCCCRC) [19].

As of 2016, Oman had 69 hospitals with over 6,400 beds, and this capacity is projected to grow at 3.1% annually. However, only two hospitals have dedicated cancer care units, with the government running 80% of healthcare facilities [20,21]. To address rising demand, the Omani government is encouraging private sector involvement in healthcare. New developments like University Medical City in Muscat and International Medical City in Salalah aim to expand services [19].

Population growth is straining the healthcare system, although 95% of Omanis live within a five-mile radius of a medical center. Due to free public healthcare, demand for cancer services is high, leading to system saturation and rising costs. While Omani citizens receive free treatment, expatriates typically rely on private hospitals, including Burjeel Hospital Oman, Muscat Private Hospital, and Aster Al Raffah Hospital. However, insurance-based and private healthcare remain limited, often excluding costly treatments like targeted therapy and immunotherapy [22].

In 2014, Oman launched Health Vision 2050, a long-term healthcare strategy aimed at expanding cancer care services, building two comprehensive cancer hospitals, and developing satellite oncology units [20-22]. This vision was recognized by the WHO as a model for strategic health planning. A key focus is decentralizing cancer care; bringing services closer to communities and homes to improve accessibility and reduce strain on hospitals [20-22].

To meet future demands, Oman must recruit 7,000 additional doctors by 2050. The country has already taken steps toward this goal, including establishing an accredited medical university and sending Omani physicians abroad for postgraduate training in Australia, Canada, the United Kingdom (UK), and the United States of America (USA) [19].

In 2025, three new hospitals were expected to open in Oman. These hospitals are Khasab Hospital in the north of Oman, Suwaiq Hospital in the middle of Oman, and Sultan Qaboos (Salalah) Hospital in the south of Oman [23]. All three hospitals will have a histopathology laboratory. This expansion in histopathology services, which involves the diagnosis of cancer, will definitely speed up cancer diagnosis and help in the early detection and treatment of cancer. However, there is a need for more comprehensive strategies, such as nationwide screening programs and community-based education initiatives, to improve early detection and reduce mortality.

While cancer diagnostic services are available in regions outside Muscat, treatment remains centralized in the capital. This geographic limitation poses a significant barrier to healthcare access for certain patients. To ensure equitable care delivery, it is imperative to expand both diagnostic and therapeutic services to remote and underserved areas.

Research and recent advances

Recent studies have investigated the importance of genetic testing and personalized medicine in breast cancer treatment. For instance, a study by Al Amri et al. [8] found that germline BRCA1/2 mutations were not over-represented in early-onset breast cancer (EOBC) cases in Oman, suggesting that Oman is not responsible for high worldwide EOBC rates. Additionally, research by Taha and Eltom [24] emphasized the role of lifestyle interventions, such as weight management and physical activity, in reducing breast cancer risk. Another study by Al-Azri et al. [11] explored the impact of cultural interventions on breast cancer awareness and screening uptake. The study found that community-based education programs significantly improved knowledge and participation in screening among Omani women. Furthermore, Al Mawali and others [22] highlighted the economic burden of breast cancer in Oman and called for increased investment in cancer care infrastructure. Moreover, Shakhman and Arulappan [25] showed that if the self-confidence of Omani women in carrying out BSE improves, they will perform BSE more often; therefore, the prevention of the development of late stages of breast cancer could be increased.

At present, the research topics of interest are breast cancer molecular subtypes, young age breast cancer in Oman, the potential role of traditional WASAM (cautery burn) therapy facilitate an early and extensive loco-regional metastasis of breast cancer, breast cancer in Kindler syndrome, stress and fatigue in breast cancer, and brain metastasis in human epidermal growth factor receptor 2 (Her-2) positive breast cancer [19].

In Oman, alongside the National Oncology Center (NOC) at Royal Hospital, which was established in 2004, the SQCCCRC was recently inaugurated in Muscat in 2021 as part of the University Medical City. This development highlights the Omani government's increasing focus on enhancing and expanding the healthcare sector, particularly in the field of cancer [26]. The center delivers healthcare services through a highly skilled multidisciplinary team (MDT), equipped with advanced medical technology and state-of-the-art information systems. Beyond medical treatment, SQCCCRC is actively involved in cancer awareness initiatives, scientific research, and training programs. The center achieved international recognition by receiving accreditation from the Joint Commission International (JCI) in June 2023 [26].

The breast cancer program at SQCCCRC offers comprehensive clinical care for patients diagnosed with or suspected of having breast cancer. An MDT comprising experts in surgical, medical, radiological, rehabilitative, and palliative care works collaboratively to provide continuous support throughout the patient's treatment journey [26]. The program adheres to evidence-based practices and follows international guidelines, offering services across various departments, including outpatient clinics, day care, and inpatient wards. Each patient's condition is thoroughly evaluated, and personalized treatment plans are developed during weekly MDT meetings [26]. Furthermore, the center has introduced the breast one-stop clinic, a specialized facility within its breast practice designed to deliver holistic breast care services. Staffed by a skilled and experienced team including breast consultants, radiologists, pathologists, and dedicated breast care nurses. The clinic collaborates closely with an MDT. This ensures that patients receive the highest quality of personalized care, tailored to their unique needs [26].

This center is expected to play a significant role in raising breast cancer awareness, enhancing early detection, diagnosis, and treatment. Additionally, it will contribute to advancing breast cancer research, deepening our understanding of the disease, and potentially reducing the morbidity and mortality rates associated with breast cancer in Oman.

The role of non-profit organizations in breast cancer awareness in Oman

Non-profit organizations play a crucial role in raising awareness, promoting early detection, and supporting patients and their families. Through education, advocacy, and community engagement, these organizations contribute to reducing the stigma associated with breast cancer and improving survival rates [11].

The National Association for Cancer Awareness (NACA) was officially registered in Oman in May 2004 and rebranded in 2014 to the Oman Cancer Association (OCA). It was the first patient voluntary support group in Oman. The OCA has partnered with various institutions across Oman, including schools, commercial centers, clubs, and associations, aiming to promote awareness and generate funds [27]. They maintain strong collaborations with both private and government sectors, particularly with the Ministry of Social Development, the MOH, the Ministry of Information, the Royal Oman Police, Muscat Municipality, and other related government bodies. Through Public-Private Partnership (PPP) initiatives, the OCA has designed and executed numerous projects effectively. Examples of these projects are mobile mammography unit (MMU), Dar Al Hanan, clinical and self-breast examination, palliative care, international cancer prevention consortium, and annual walkathon [19,27].

The key contributions of non-profit organizations may take many forms. Public awareness campaigns that aim to educate women about breast cancer symptoms and the importance of regular screenings are one of them [11]. In addition, free screening programs via the MMU or collaborating with hospitals to provide free mammograms, particularly for low-income women, is a key role of non-profit organizations [19]. These organizations also play a critical role in offering psychosocial support and financial assistance for treatment, if required. Furthermore, they conduct workshops to enhance early diagnosis skills among medical professionals and work with the government to improve cancer care policies and funding [27].

Strengthening partnerships between non-profits, government agencies, and the private sector is essential to enhance breast cancer awareness and care in Oman.

## Conclusions

Breast cancer remains a major public health challenge in Oman, with rising incidence rates and significant cultural barriers to early detection. While progress has been made in improving awareness and treatment, more efforts are needed to address the unique challenges faced by Omani women. Future research should focus on understanding the cultural and social determinants of breast cancer and developing targeted interventions to improve outcomes.

Furthermore, there is a critical need for governmental policy initiatives to decentralize cancer treatment services beyond the capital and enhance funding for cancer research, with a particular emphasis on breast cancer.
